# On the Action of Mercurial Ointment and the Vapor of Mercury

**Published:** 1851-01

**Authors:** 


*On the Action of Mercurial Ointment and the Vapor of Mercury.*—The *American Journal of Pharmacy*, for January, 1851, contains a paper from F. Von Bærensprung, M.D., in which is recorded a series of interesting experiments on the action of triturated mercurial preparations, and mercurial vapor. The experiments were very skilfully conducted, and the results are useful to practitioners of medicine.

RESULTS. 1st.—*It is first established that the metallic mercury in mercurial ointment does not pass into the body as such.*

2d.—*It is proved that mercurial ointment contains protoxide of mercury in addition to metallic mercury and fatty matter.*

3d.—*The greater part of the mercury in the blue ointment is perfectly inactive. One grain of the oxide of mercury to ℥j of fatty matter is equal, in strength and action, to the blue mercurial ointment. Hence its great superiority.*

These results apply also to the other preparations made by the trituration of metallic mercury.

Results of experiments with mercurial vapor: 1st.—It is proved that mercury does not penetrate animal membranes even in the gaseous state; hence it cannot be taken up in the living body.

Vapors of mercury first produce inflammation of the lungs, subsequent oxidation and absorption take place, and mercurialism commences.

